# Lessons Learned as President of the Institute for Systems Biology (2000–2018)

**DOI:** 10.1016/j.gpb.2018.02.002

**Published:** 2018-03-02

**Authors:** Leroy E. Hood

**Affiliations:** 1 Providence St. Joseph Health, Seattle, WA 98057, USA; 2 Institute for Systems Biology, Seattle, WA 98109, USA

**Keywords:** 21st-Century medicine, Systems medicine, Scientific wellness, P4 healthcare, Systems biology, Personal, dense, dynamic data clouds

I stepped down as president of the Institute for Systems Biology (ISB) on Jan 1, 2018. As I think about my 17-year term as President, I am astounded at how much I have learned, not only about science but also about, among other things, what it takes to build a unique world-class institution.

## The beginnings

I came to the non-profit, independent ISB from 30 years in academia. For 22 years, I was a faculty member of Division of Biology at the California Institute of Technology (Caltech), the last 10 years as chair of the division. Following Caltech, I spent 8 years at the University of Washington (UW) Medical School as founder and chair of the Department of Molecular Biotechnology (MBT), the first cross-disciplinary biology department to my knowledge in the world.

I resigned from the UW in December 1999 to launch Institute for Systems Biology (ISB) in mid-2000—the first institute dedicated to the nascent discipline that I coined as “systems biology.”

I had been thinking about biological complexity for decades. My colleagues and I began to create new technologies and strategic approaches to address this complexity. We developed at Caltech and MBT automated biological instrumentation that led to high-throughput biological measurements and the data that initiated the big data era for biology. The automated DNA sequencer enabled the human genome program. I played a role in advocating and helping to execute this program, commercializing a genomic approach to drug discovery (Darwin Inc.), and applying the fruits of genomic discovery to human medicine. The Human Genome Project was biology’s first big science project and it transformed many different aspects of biology and medicine as well as our understandings of Darwinian evolution and human migrations.

I started to conceptualize systems biology in the late 1980s and even wrote several unsuccessful grants on this topic at that time. Over the next decade, my thinking matured about how to define systems biology and employ it to unravel biological complexity in a global or holistic manner, quite distinct from the traditional approach of studying biology one gene or one protein at a time.

The complexity of the organizational structure of a large state university made it difficult to introduce this new discipline—requiring the integration of biology, technology, and computation, which was so different from the pre-existing biological disciplines. I realized that comprehensive systems biology could only emerge effectively from a new organizational structure. So, I founded ISB with a vision of systems biology and a sense of what was required to build an appropriate research environment, a supportive culture and the necessary scientific leadership and expertise. We started with commitments to bring knowledge to society, to mature ISB rapidly, and to achieve simplicity in administrative organization and decision making.

## The vision and execution of systems biology at ISB

**Figure 1:**
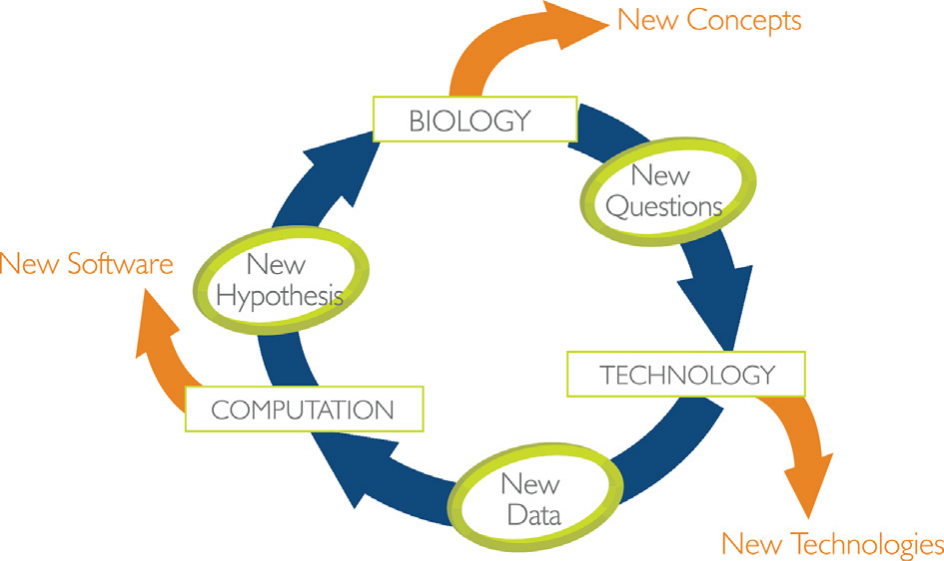
**The cross-disciplinary philosophy of ISB—biology drives technology drives computation** This engine of discovery also leads to innovation through company creation with new technologies, new software and new concepts.

Systems biology takes a global and holistic approach to addressing biological complexity—where biology drives the pioneering of relevant technologies and the resulting data drive the invention of computational tools (Figure 1). This vision has fueled the on-going evolution of ISB. Critical elements of systems biology are listed as follows.

### Cross-disciplinary research environment

Systems biology must be embedded in a cross-disciplinary environment with biologists, chemists, computer scientists, engineers, mathematicians, physicists, and physicians. Our fundamental mantra is that leading-edge systems biology should drive the development of relevant technologies, and these, in turn, should push the creation of the necessary computational tools for handling the relevant data (often big data). These approaches have transformed our understanding of biological complexity.

### Collaborative culture

Systems biology must have an appropriate cultural environment to foster and encourage independence, creativity, excellence, interactions, respect, passion, determined optimism (to get through the difficult times), and encouragement to think big enough to “invent the future.”

### Scientific leadership and expertise

To rapidly build an outstanding scientific environment, I felt that at least three outstanding senior scientists were necessary to catalyze rapid outstanding faculty recruitment. I persuaded Alan Aderem, an excellent immunologist, and Ruedi Aebersold, a global leader in protein chemistry and proteomics, to join ISB. The three of us were the co-founders of ISB and our combined scientific strengths and reputations quickly attracted a world-class faculty and staff. The faculty of ISB beautifully reflect the essence of systems biology—with interests in biology, medicine, technology, computation, and theory (see below).

### Transfer of knowledge to society

The transfer of knowledge to society is fundamental to ISB’s mission. We initially focused on two areas in this regard—science education for K-12 students, and innovation and company creation. The Valerie Logan Center for Education was established to enable professional teacher training with access to high quality Science, Technology, Engineering, and Mathematics (STEM) education. We transformed K-12 science education in Seattle schools and more recently have engaged almost half the school districts in the state in STEM education through professional teach training.

I had founded or co-founded 8 companies, including Amgen and Applied Biosystems (ABI commercialized the 4 instruments my lab co-developed at Caltech), prior to the initiation of ISB and thus had a great deal of experience in this realm. ISB has created 8 companies in its 17 years of existence, several of which have brought millions of dollars to ISB for research support. Hopefully in the future one of these companies will do extremely well and provide a substantial endowment for ISB.

### Nimble organizational structure

ISB needed a simple administrative organization that could adapt quickly to new opportunities and could rapidly make important (and even less important) decisions. We achieved that.

Bringing these concepts to life at the new Institute began immediately. In some areas, it was easy. In others, we debated considerably about the issues. Should we have K-12 education as a central feature of ISB? It did. Should innovation and company creation become a major pillar of ISB? It did. One particularly vigorous debate was whether to seek grants from the Department of Defense. We did. With some of the ideas related to systems biology and systems medicine, it turned out to be an ongoing evolution of conceptual thinking moving in different directions driven by different faculty members. The applications of systems biology to biology and medicine commenced immediately (see later).

Many new surprises and marvelous opportunities came from the creation of ISB.

## Transformational events for ISB

To put ISB in perspective, there were four major transformational events during its evolution.

**Figure 2:**
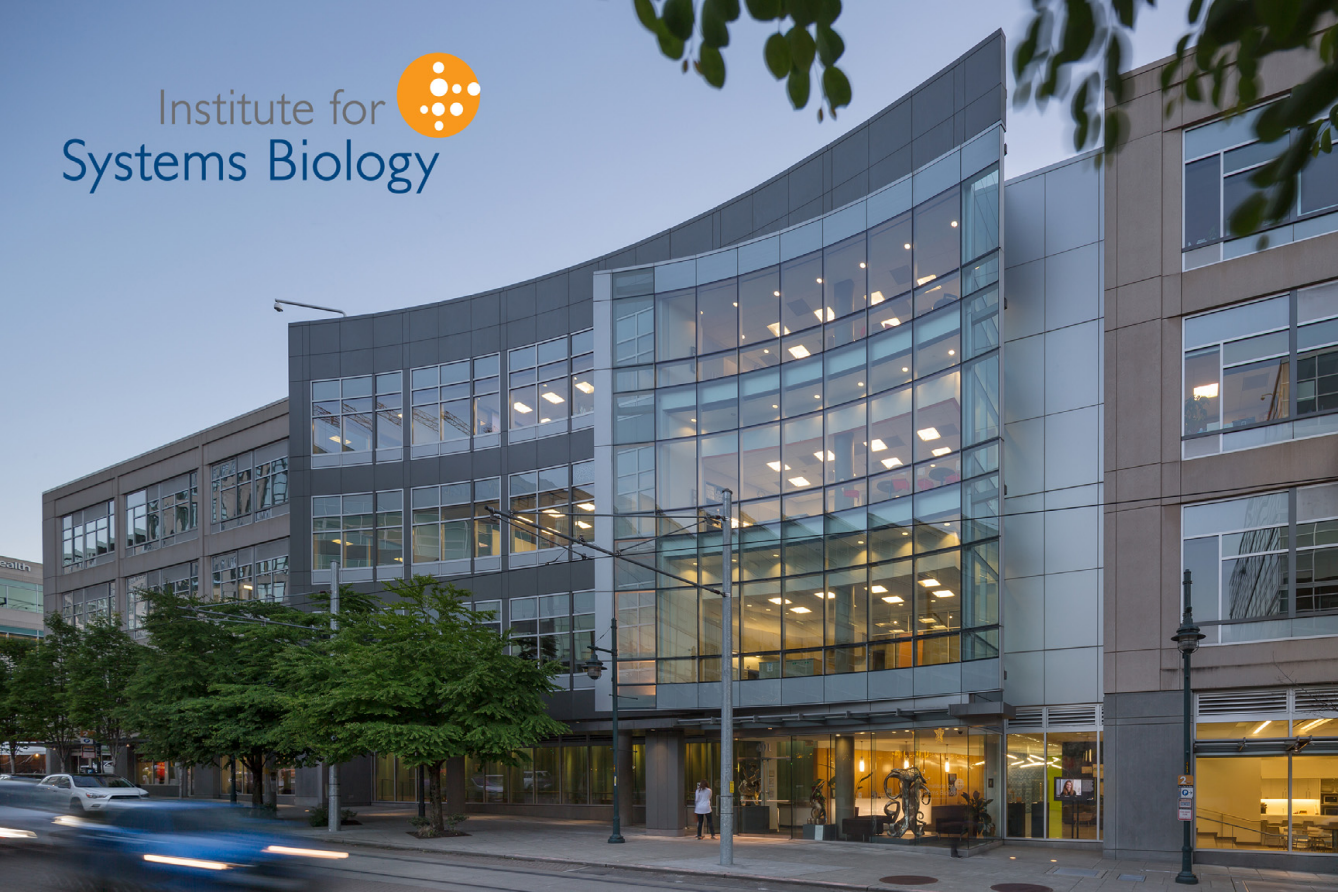
**A photo of the ISB building located at the South Lake Union**

First, our three moves, each time expanding to a larger facility, which created new opportunities: (1) MBT at UW – to the very cramped quarters at Roosevelt in the University District in 2000; (2) Roosevelt – to North Lake Union location in 2001; and (3) North Lake Union – to South Lake Union site in 2011 (Figure 2). Each expansion allowed us to reintegrate groups we had necessarily located at dispersed sites, enabled future growth and new infrastructure, and inspired us to recommit to the ongoing evolution of systems thinking.

Second, the Center for Systems Biology at ISB is one of 11 national systems biology centers funded by the National Institute for General Medical Sciences. Established in 2006 and funded through 2017, the ISB Center provides resources for the integration of big science projects, the exploration of new scientific directions, the support of activities to create a systems-biology-driven culture, and the training of students at all levels. The Center provided the glue that really facilitated interactions and the evolution of a systems-driven culture. John Aitchison did a marvelous job as the most recent director.

Third, the strategic partnership with Luxembourg brought ISB $100 million, $20 million dollars per year from 2008–2013, to invent about 10 technologies and strategies for systems biology (see discussion below).

Fourth, in April of 2016, ISB affiliated with the large, Seattle-based, non-profit healthcare system —Providence St. Joseph Health (PSJH). ISB has become the research arm for PSJH and I became its chief scientific officer. This affiliation provides the opportunity to bring systems-driven medicine to the US healthcare system (see discussion below), which will transform health care to a proactive force, focused on optimizing individual’s wellness and identifying the earliest opportunities to reverse or even prevent disease.

## Launching ISB was challenging

We needed about $20 million to build a functional systems-biology-driven infrastructure for science at ISB. Unfortunately, the Institute was launched at just about the time of an enormous economic downturn. I had planned to ask several of my affluent friends for support, but I discovered that they were not eager to respond to a large philanthropic request, given that they had just lost a significant fraction of their investment portfolios. Moreover, several had questions about whether the newly-minted systems biology was really an appropriate vision on which to found an institute.

Fortunately, my wife Valerie Logan and I could personally provide substantial early investments in ISB that continued throughout its history. In addition, Roger Perlmutter through Merck gave a $4 million gift in the early days of ISB. We also received a series of smaller gifts from various philanthropists, and several foundations (*e.g.*, the Murdock Foundation) were very generous. Once it was clear that we had adequate support, Alan Aderem and Ruedi Aebersold joined me from the UW (mid-2000) and ISB was on its way. Later, Bill Gates and Bill Bowes (a San Francisco venture capitalist-turned philanthropist, who started Amgen and Applied Biosystems with me) contributed almost $30 million to the Institute.

## New technologies and systems-driven strategies were transformational

From the beginning, I realized an important imperative for ISB was to invent relevant new technologies for systems biology to explore new areas of data space. ISB has been highly successful in pioneering new technologies: genomics with an instrument from the Hood lab analyzing single RNA molecules that became an ISB spinout company, Nanostring Technologies; proteomics with the application by Moritz and Aebersold of highly sensitive targeted mass spectrometry-based proteomics (*Nature’s Technology of the Year, 2014*); and novel applications of single-cell analyses to human development and cancer by Huang and Hood.

As ISB progressed, the importance of system-driven strategies—one or more technologies connected by a computational platform for high throughput data generation—became evident. These included (1) personal, dense, dynamic data clouds to characterize individual wellness and disease; (2) family genome sequencing to identify disease genes; (3) systems-driven blood protein biomarker discovery with mass spectrometry to generate biomarkers that distinguish normal from diseased individuals (lung cancer, preterm birth, glioblastoma, *etc.*); (4) organ-specific blood protein analyses for the identification of diseased organs and important blood biomarkers (*e.g.*, Lyme disease and liver diseases); and (5) drug target discovery strategies employing the computational analyses of disease-perturbed biological networks.

## Evolving visions have driven the emergence of systems biology and the initiation of systems medicine (P4 medicine)

Our thinking about systems biology has evolved considerably over the past 17 years—very much driven by new technologies and systems-driven strategies that opened new dimensions of data space (for humans and other organisms). For example, single-cell analyses contribute unique opportunities to deconvolute biological complexity. Indeed, the idea that complexity can be attacked at the four organizational levels of biological information in living organisms—single molecule analysis, single-cell analysis, the analysis of individual or single organs, and the analysis of the whole individual organisms—has been pioneered by ISB. We have developed technologies, strategies, and the integrative computational tools that encompass each of these four levels, with the exploration of each of the levels giving us new opportunities for understanding complexity and deciphering more effectively human disease.

**Figure 3:**
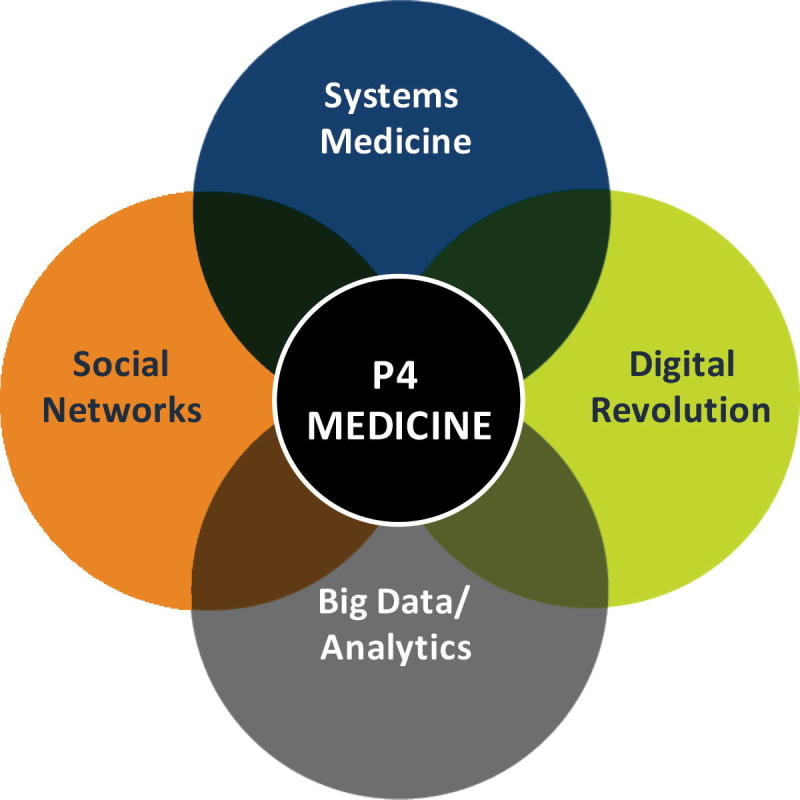
**P4 medicine is partially defined by the convergence of systems medicine, digital revolution (of self-measurements), big data, and social networks**

The concepts that emerged from systems approaches to disease have really evolved at ISB, all initially formulated in the first few years of our existence. *Systems medicine* is the systems approach to disease (including new technologies and systems-driven strategies). This concept led to the idea that healthcare should be predictive, preventive, personalized, and participatory (P4). Moreover, we realized that *P4 healthcare* should have two major thrusts—wellness and disease, whereas wellness is almost entirely ignored by the conventional 20th-Century Medicine (Figure 3).

We further defined quantitative wellness by generating dense, longitudinal data clouds for individual humans that, when analyzed, led to actionable possibilities that could improve wellness and avoid or ameliorate disease, enable novel insights into mechanisms of wellness and disease, and provide new approaches to biomarker discovery and the identification of drug target candidates. We termed this quantitative wellness as “scientific wellness”.

The integration of a systems approach to disease, systems medicine, P4 healthcare and scientific wellness, collectively constitute the essence of the *21st-Century Medicine*, which will improve the quality of healthcare, lead eventually to a reversal of all chronic diseases at their earliest transition points, and enormously decrease the cost of healthcare. These conceptual formulations, including systems biology, have continued to evolve with the emergence of systems-driven technologies and strategies, as well as computational tools and revolutionary new technologies (*e.g.*, single-cell analyses). This evolution of thinking about systems biology and systems medicine has led to four ISB faculty to write a textbook on this topic (see below).

## Big science is essential for deciphering complex biological systems

Big science — which is predicated on the idea that most interesting biological problems are complex and require the coordinated organization of many different scientific disciplines to tackle their complexity — has played an incredibly important role in ISB’s research agenda.

HGP was biology’s first big science initiative. It required the coordination of teams of researchers from around the world engaged in sequencing DNA, and developing the relevant technologies and computational tools necessary for delivering the first complete sequence of the human genome in 2003. It brought, for the first-time, computer scientists and engineers into biology in large numbers. This project transformed the whole landscape of biology and it is beginning to impact medicine.

ISB has taken on a series of big science problems—preterm birth, the development of the technologies and systems-driven strategies of systems medicine, scientific wellness, the Cancer Genome Atlas (TCGA) project and the sequence analysis of thousands of human genomes to characterize their general features and to correlate genetic variation with wellness or disease phenotypes. Each of these big science projects has required senior leadership and expertise, an implementation plan with timelines, the integration of both science and engineering techniques, and generation, correlation and interpretation of vast quantities of data. Often, we must deal with ethical and regulatory issues as well.

Big and small science are synergistic. Big science creates many opportunities for small science. Small science generally is a lab of 10 or fewer focused on a single or very limited set of problems. Big science creates hypotheses that can be tested by small science, as well as powerful new technologies and computational tools for facilitating small science (*e.g.*, high-throughput DNA sequencing) and the computational tools for data analysis.

## Strategic partnerships are often critical in helping solve big problems

Big science quite naturally led to the emergence of strategic partnerships—which could bring together missing scientific and engineering talent, new technologies and strategies, collaborators to tackle difficult problems, and in some cases, significant financial resources. All strategic partnerships require strong leadership and a clear science vision. I became convinced that bringing systems biology to institutions that desired 21st-century science could provide that vision and at the same time create resources for ISB to invent the future of healthcare.

By the year 2005, ISB had pioneered the concept that healthcare should be P4 medicine/healthcare. ISB sought partners who would participate in developing the systems-driven strategies and technologies of P4 medicine and eventually bring them to healthcare systems.

In 2005, I began exploring the possibility that other countries or regions might be interested in launching their own Institutes for Systems Biology and providing ISB with resources for carrying out this task. I started in Israel at the request of a fundraiser who was convinced he could raise $200 million, which would fund the building of an Institute in Israel, and provide $100 million for ISB. I went to Israel twice and visited all the major academic institutions, making the pitch for a systems biology institute. Beersheba University had an outstanding president who was very enthusiastic about this possibility and even initiated the design of a building to house the newly proposed Systems Biology Institute. Unfortunately, the fundraiser was not successful and my first attempt at a significant strategic partnership ended in failure. In the next three years, I made similar pitches to agencies in Ireland, Korea, and Alberta—and for distinct reasons each time the pitch failed. I had accumulated an enormous amount of experience on strategic partnership failures. After the Israel failure, the ISB board chair strongly suggested that I give up this ‘fruitless” search. But I persisted—determined optimism is essential for bringing new ideas and paradigms to reality.

In 2007, I met the Minister of Economy for the Grand Duchy of Luxembourg—who was focused on diversifying its economy away from a 90% dependence on financial services. His goal was to transform Luxembourg into a center of excellence in personalized medicine by investing heavily in the biotechnology sector. He invited ISB to submit a proposal to facilitate this objective.

**Figure 4:**
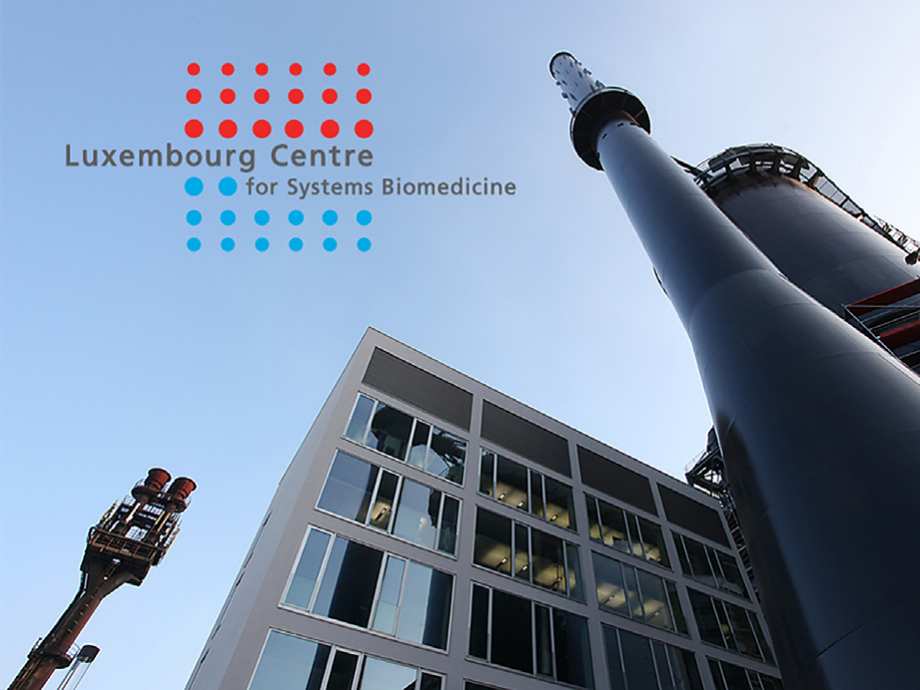
**A photo of the Luxembourg Center for Systems Biomedicine—a center ISB played a fundamental role in creating**

We proposed to build a Luxembourg Center for Systems Biomedicine (LCSB) at the newly-formed University of Luxembourg, where ISB would recruit the director, help recruit faculty, and train 11 postdoctoral fellows in the science and technologies of ISB who would return to LCSB. ISB faculty would collaborate with LCSB on science, technologies and computational biology and we also would create a network of outstanding, interacting scientists across the European Union (EU) and United States (USA) to assist in this effort. We succeeded in each of these endeavors and today LCSB is one of the leading life sciences institutions in the EU (Figure 4). This was done in about three years with the major contributions from ISB—and it could never have been done without ISB’s leadership. In return, we proposed that Luxembourg provide $100 million over five years for ISB to invent the technologies and strategies of P4 medicine. During the Luxembourg partnership, ISB pioneered 10 of these technologies and strategies.

**Figure 5:**
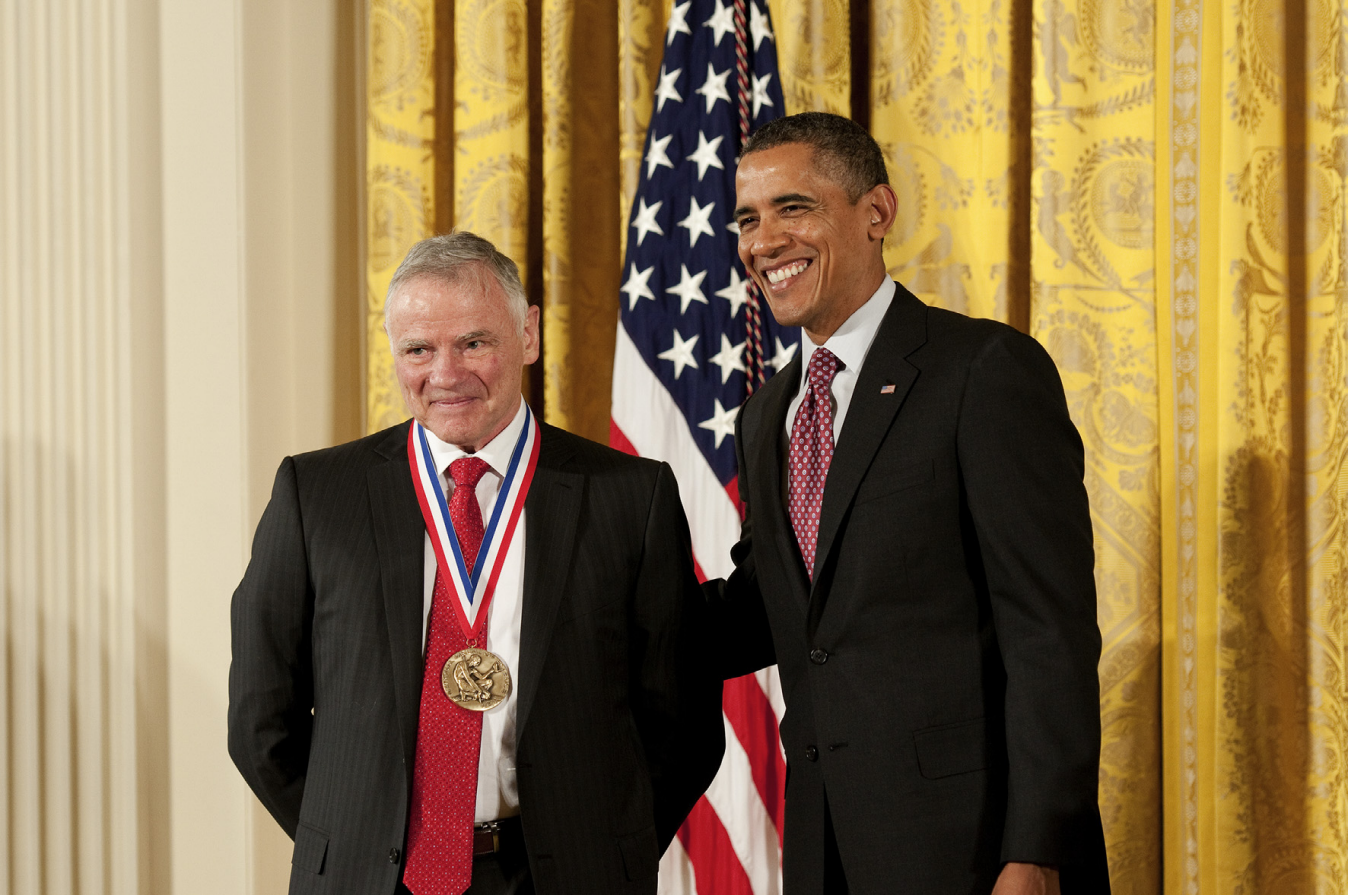
**A photo of Leroy Hood and former President Barack Obama during a 2013 White House ceremony awarding the National Medal of Science**

This success with Luxembourg placed systems medicine at a tipping point in 2014, where we could clearly see how we could implement many aspects of P4 healthcare in healthcare systems, if we could find willing partners.

## Scientific wellness and the emergence of Arivale

We then decided to pioneer and quantify scientific wellness and launched a pilot project with 108 individuals using personal, dense, dynamic data clouds over nine months. The 108 participants in this project were so enthusiastic about its outcomes that in 2015 we launched Arivale, a company that brings scientific wellness to consumers and industrial enterprises. Arivale now has 3500 participants. ISB is partnering with Arivale using the de-identified data from these individuals for computational analyses of human wellness, human disease, and the transitions from wellness to disease for most common chronic diseases. The exciting idea is to get biomarkers for these transition points and then to use them to pioneer therapies using systems technologies and strategies to reverse chronic diseases at their earliest transition, before they ever manifest themselves as an irreversible disease phenotype—this will be the preventive medicine of the 21st century. This is a marvelous example of how a spin off company can play a major role in helping ISB realize and validate a big scientific objective—scientific wellness.

### P4 medicine/healthcare and precision medicine

Systems approaches to medicine at ISB from 2000–2004 led to the concept of a P4 healthcare. The definition of P4 medicine arises from the convergence of 5 social and medical thrusts: it embraces the concepts of systems medicine and scientific wellness; it utilizes the striking opportunities of digital health; it capitalizes on big data and its analytics; and it takes advantage of diverse social networks for education, advocacy, and recruitment. Moreover, it also focuses on the individual assessing his/her genetic and lifestyle/environmental contributions to health with his/her own data clouds and with help from coaches or physicians make his/her own decisions about his/her health. Precision medicine, born with Obama’s State of the Union Address in 2015, employs big data (mostly from genomics) and digital health to target disease. For example, one of the most “successful” examples of precision medicine is the DNA sequencing of tumors to identify cancer driver mutations that might have complementary drugs—which can then be employed to attack the tumor. One irony about precision medicine is that medicine is mostly not precise—indeed, precision does not define at all what type of medicine it represents. The contrast between P4 medicine (healthcare) and precision medicine is striking. P4 medicine is systems driven and embraced the study of wellness and disease in the context of the individual. In addition, the four Ps describe the ideals of what we want our healthcare systems to provide—predictive, preventive, personalized, and participatory. P4 is the very essence of 21st-Century Medicine. In contrast, precision medicine focuses entirely on disease—employing the reductionistic approaches of 20th-Century Medicine, while adding the power of big data and the devices of digital health. Indeed, one would argue that the personal, dense, dynamic data clouds that scientific wellness pioneered in 2014 represent the very essence of what precision medicine of 2015 should be.

## ISB-PSJH affiliation opens the possibility of bringing P4 healthcare and 21st-Century medicine to a large healthcare system

Late in 2015, Rod Hochman MD, CEO of the large non-profit Providence Health Systems (now PSJH), approached me with a striking proposition. He suggested that ISB become the research arm of Providence and that I serve as Providence’s Chief Scientific Officer. He understood the power of scientific wellness and was sympathetic to the vision of 21st-Century Medicine. In a moment, I realized that this was a pathway to bringing P4 healthcare to the US healthcare system. Indeed, over the preceding 5–6 years, I had approached 6 top-ranked academic medical institutions—including my alma mater, Johns Hopkins University. I generally pitched the idea of ISB helping the institution build a Systems Medicine Center as we did with Luxembourg. Skepticism, scientific silos, and a commitment to 20th rather than 21st Century Medicine objectives in various combinations led all to decline my offers. I don’t think any of them realized how profoundly systems thinking is changing medicine and opening the way for 21st Century Medicine. So, it was exhilarating to find that a large non-profit community healthcare system could enthusiastically embrace P4 healthcare and systems medicine. In April of 2016, ISB and Providence signed an affiliation agreement to connect ISB’s systems approaches to medicine with the clinical expertise at PSJH to begin to shift health care delivery from a predominant disease focus to a wellness focus with powerful strategies for prediction and prevention.

Providence did not have a formal, overarching research organization in place prior to the affiliation—although it did have outstanding scientist-clinicians at many of its 50 hospitals and two cancer research centers. Over the last 18 months, I have given 25 lectures for Providence leaders, scientists and clinicians introducing the idea that systems-driven research, properly supported, will enable PSJH to invent the future of healthcare. I created a Scientific Advisory Counsel of 23 members drawn from PSJH and ISB scientists and clinicians to develop a strategic vision for research in PSJH.

Nathan Price and I are creating a Center for Translational Systems Medicine (CTSM)—to enable a powerful interface between ISB and PSJH. CTSM has identified 10 translational pillars (clinical trials using systems approaches and personal, dense, dynamic data clouds), a technology platform to create these data clouds, specialty clinics for scientific wellness and Alzheimer’s disease, and an educational program to bring the vision of 21st-Century Medicine to students, patients, MDs in training, and healthcare professionals.

PSJH is the first (and currently the only) healthcare system beginning to practice systems-driven 21st-Century Medicine. Together PSJH and ISB will be among those inventing the future of healthcare—both nationally and eventually globally.

## ISB faculty are diverse in skills and interests

The ISB faculty are a remarkable collection of individuals with broad skills and unique orientations.

### John Aitchison

An outstanding systems cell biologist with an interest in big and challenging problems, such as nuclear transport. He has also developed powerful technologies that focus on cells.

### Nitin Baliga

A systems microbiologist and computational biologist with broad and diverse interests in the environment and medicine, who has pioneered marvelous tools to reveal the secrets of biological networks. He has also pioneered some remarkable programs for the education of K-12 students in systems biology and food production.

### Sui Huang

A marvelous conceptual thinker with an interest in using single-cell analyses to decipher the complexities of biology and disease. He is demonstrating that many of our previous convictions about cancer are totally wrong. He is marrying theory with good biology.

### Robert Moritz

A skilled protein chemist with a broad and comprehensive focus on developing proteomic technologies to define the proteomes of cells, organs, and organisms to, in the case of humans, search for biomarkers and even drug and vaccine targets.

### Nathan Price

A superb computational biologist with an interest in metabolism, transcriptional networks, scientific wellness, and 21st-Century Medicine. He is pioneering the development of the CTSM to serve as an interface between ISB and PSJH.

### Jeff Ranish

An accomplished protein chemist working on cross-linking regents for protein complexes that are helping delineate the structure of complex, multi-sub-unit proteins and one day will even delineate biological networks.

### Ilya Shmulevich

A brilliant engineer with an interest in signal processing, large-scale computing and large-scale modeling, whose skills have been focused on largely cancer through TCGA project.

### Naeha Subramanian

A systems immunologist and molecular biologist interested in immunity and its impact on infectious diseases such as Lyme disease. Naeha has a wonderful understanding of the depths of modern immunology.

## Scientific papers

Perhaps what is most exciting about what I have learned at ISB is the science, so I also want to highlight some of the papers that I have co-authored during my ISB tenure that have made fundamental contributions to systems biology, disease, technology, and healthcare:

(1) A review which defined the fundamental principles of systems biology in 2001 that remain valid today [1].
(2) The creation of the Cytoscape algorithm in 2001—a graphical construction of networks from biological data—one of the most widely used algorithms today in computational biology. Cytoscape was pioneered by Trey Ideker, who to this day continues to lead its evolution [2].
(3) A study in 2001 of how dynamical networks explain yeast galactose metabolism—this was the first application of a systems approach to explain a biological mechanism [2].
(4) A study in 2007 of how dynamical network analyses allows one to predict the responses of a single celled organism, halobacterium, to changes in its environment—a milestone in predictive systems thinking pioneered by Nitin Baliga [3].
(5) The spinoff of a concept about the single molecule detection of mRNA (microRNA) molecules that led to the creation of Nanostring, Inc. [4].
(6) A study in 2009 of how dynamical disease-perturbed networks explain prion-induced neurodegeneration in mice—which opened an entirely new approach to demystifying disease [5].
(7) In 2010 and in 2016, we pioneered family genome sequencing which allows one to identify disease-genes—for both simple diseases and complex diseases like bipolar disorders [6], [7].
(8) A series of papers conceptually and practically defining systems medicine, P4 healthcare, and scientific wellness from 2003 through 2017 [8], [9], [10], [11], [12], [13].
(9) Approaches to the use of nucleic acids as disease biomarkers [14], [15]. Several papers on the use of systems approaches to protein blood biomarker discovery, which allowed one to create diagnostic lung cancer (Integrated Diagnostics, Inc.) and preterm birth (Sera Prognostics, Inc.) protein blood panels—tests that are being used in clinical practice today from 2014 through 2017 [16], [17], [18].
(10) Quantifying scientific wellness with personal, dense, dynamic data clouds, which when analyzed lead to actionable possibilities that allow one to optimize wellness and avoid disease (2014–2017). These data will also transform how pharma, diagnostic companies, biotechnology companies, and nutrition companies will carry out their work in the future [19], [20], [21], [22].
(11) The 2016 ISB Annual Report on how 21st Century Medicine, comprised of P4 healthcare, systems medicine, and scientific wellness will transform healthcare in the future [23].
(12) The analysis at the single cell level of the differentiation of induced pluripotent stem cells to cardiomyocytes demonstrated many of the principles of dynamical systems theory—a complex landscape representing the various pathways that cells could traverse, bifurcation points, and attractors—this enabled one to compress a remarkable amount of data into a simple model of this process—one that suggested how the process could be manipulated experimentally to produce high yields of cells of a given type [24].
(13) An analysis of the benefits of HGP [25].
(14) A text book on Systems Biology and Systems Medicine with ISB faculty members Sui Huang, Nathan Price, and Ilya Shmulevich that has been submitted to a publishing company and is scheduled to come out at the end of 2018.

## Coda

As you can see, ISB has been a far more exciting adventure than I ever could have imagined. It changed my entire perspective on science and medicine, and how we can transform the future. And the future today looks even brighter than this very exciting past described in this article. We will change the healthcare system to 21st Century Medicine (the big question is how long this will take). I continue to learn from this adventure and I continue to have fun in all dimensions of my life. Those are the secrets of eternal youth (plus exercise!).
